# A high-tech wreck: HIPAA roadblocks to texting patients

**DOI:** 10.1038/s41372-024-02131-x

**Published:** 2024-10-01

**Authors:** Jonathan Fanaroff

**Affiliations:** Department of Pediatrics, Case Western Reserve University, Cleveland, OH USA

**Keywords:** Patient education, Health policy

Protecting the private and confidential information entrusted to health care professionals is a core principle dating back to the Hippocratic Oath. At the same time, effective health communication is essential to build trust and allow patients and families to understand and participate as true members of the team. Although it is not often discussed, there can be a tension between legal security regulations designed to protect confidentiality and the ability to effectively communicate with families.

The main source of this tension in the United States is related to the Health Insurance Portability and Accountability Act (HIPAA). At the dawn of the electronic medical record era, Congress was concerned about security and so in 1996 passed the Health Insurance Portability and Accountability Act (HIPAA). Under HIPAA, there are potential civil and criminal penalties for failing to protect specified health information. These are not merely theoretical punishments. Since inception there have been several hundred thousand complaints of HIPAA violations and the Office for Civil Rights has levied several high profile multi-million dollar fines for violations, including a record $16 million dollar fine against Anthem, Inc. in 2018.^1^ This has understandably made health care institutions highly risk averse. But have they become too risk averse, and at what cost?

In this issue of *Journal of Perinatology*, Lindsey et al. discuss the legal barriers they encountered when attempting to use text messaging-based digital health interventions as part of a randomized controlled trial specifically designed to improve maternal and neonatal outcomes in marginalized communities.^2^ While developing the well-named POPPY trial, standing for Providing an Optimized and emPowered Pregnancy for You, the goal was to use text messaging as a quick and accessible way to deliver pregnancy-related health care information to patients living in the 4^th^ or 5^th^ Quintile of the Area Deprivation Index. Patients from these areas often lack stable access to the smartphones and internet that are often used to interact with health care systems. They do, however, almost always have access to text messaging, which is widely accessible and indeed one of the most common forms of communication. Mobile phone users in the United States sent approximately 2 trillion text messages in 2021 alone.^3^

Technology has had a revolutionary impact on almost all aspects of society in the past few decades, and provided a number of benefits to patients and healthcare professionals. But there are concerns that there is unequal distribution of these benefits leading to a ‘digital divide.’ Indeed, to the extent that technology has the ability to improve health outcomes, lack of access to technology can lead to an even greater gap as “populations that have poorer health outcomes continue to have poorer health outcomes despite technological improvements.”^4^ The POPPY trial envisioned bridging this divide by using simple text messaging to improve access to quality health information and improve both the care and experience during pregnancy and after delivery. Due to HIPAA concerns, however, the investigators were forbidden from using simple text messaging and instead required to implement security measures involving increased complexity, additional clicks, and a secure browser requiring internet access. Unsurprisingly, engagement with the promising intervention declined, in part due to the fact that access was now denied to patients with a mobile phone that had texting ability but no internet capability. An intervention specifically designed to improve inclusion and accessibility was now less accessible and less inclusive.

As the authors recognize, it is a “formidable challenge” to appropriately balance effective communication with privacy. Possible solutions will involve healthcare institution’s developing and sharing innovative approaches. The Department of Health and Human Services, which regulates HIPAA, must be involved as well. Most important is involving, and listening to, affected patients who will have crucial opinions on effective solutions.

Patients want and expect their health information to remain protected and confidential, and it is essential that pregnant women feel comfortable disclosing highly sensitive and personal information to receive optimal maternity care. At the same time, a key purpose of HIPAA was to improve “the efficiency and effectiveness of the health care system.”^5^ Lindsey et al. are to be commended for sharing the barriers they faced when attempting to use texting to improve patient outcomes, as it is only when problems are shared that solutions can be developed.

## Footnotes

1 Department of Health and Human Services Press Office. Anthem Pays OCR $16 Million in Record HIPAA Settlement Following Largest U.S. Health Data Breach in History. 10/15/18.

2 When HIPAA Hurts: Legal Barriers to Texting May Reinforce Healthcare Disparities and Disenfranchise Vulnerable Patients [need additional citation information].

3 https://www.statista.com/statistics/185879/number-of-text-messages-in-the-united-states-since-2005/.

4 Saeed SA, Masters RM. Disparities in Health Care and the Digital Divide. Curr Psychiatry Rep. 2021 Jul 23;23(9):61.

5 Health Insurance Portability and Accountability Act of 1996, Public Law 104-191 Section 261 Purpose.

